# Use of probiotics in patients with chronic kidney disease on hemodialysis: a randomized clinical trial

**DOI:** 10.1590/2175-8239-JBN-2022-0021en

**Published:** 2022-09-12

**Authors:** Érica Maria Rodrigues de Araújo, Gdayllon Cavalcante Meneses, Antônio Augusto Ferreira Carioca, Alice Maria Costa Martins, Elizabeth De Francesco Daher, Geraldo Bezerra da Silva

**Affiliations:** 1 Universidade de Fortaleza, Programa de Pós-Graduação em Ciências Médicas, Fortaleza, CE, Brazil.; 2 Universidade de Fortaleza, Curso de Nutrição e Programa de Pós-Graduação em Saúde Coletiva, Fortaleza, CE, Brazil.; 3 Universidade Federal do Ceará, Programas de Pós-Graduação em Farmacologia e Ciências Farmacêuticas, Fortaleza, CE, Brazil.; 4 Universidade Federal do Ceará, Faculdade de Medicina, Programa de Pós-graduação em Ciências Médicas, Departamento de Medicina Clínica, Fortaleza, CE, Brazil.

**Keywords:** Renal Insufficiency, Chronic, Probiotics, Gastrointestinal Microbiome, Inflammation, Biomarkers, Insuficiência Renal Crônica, Probióticos, Microbioma Gastrointestinal, Inflamação, Biomarcadores

## Abstract

**Introduction:**

Supplementation with probiotics for patients with chronic kidney disease (CKD) may be associated with decreased systemic inflammation.

**Objective:**

To assess the impact of oral supplementation with probiotics for patients with CKD on hemodialysis.

**Method:**

This double-blind randomized clinical trial included 70 patients on hemodialysis; 32 were given oral supplementation with probiotics and 38 were in the placebo group. Blood samples were collected at the start of the study and patients were given oral supplementation with probiotics or placebo for three months. The probiotic supplement comprised four strains of encapsulated Gram-positive bacteria: *Lactobacillus Plantarum A87, Lactobacillus rhamnosus, Bifidobacterium bifidum A218* and *Bifidobacterium longum A101*. Patients were given one capsule per day for 3 months. Blood samples were taken throughout the study to check for inflammatory biomarkers. Non-traditional biomarkers Syndecan-1, IFN-y, NGAL, and cystatin C were measured using an ELISA kit, along with biochemical parameters CRP, calcium, phosphorus, potassium, PTH, GPT, hematocrit, hemoglobin, glucose, and urea.

**Results:**

Patients given supplementation with probiotics had significant decreases in serum levels of syndecan-1 (239 ± 113 to 184 ± 106 ng/mL, p = 0.005); blood glucose levels also decreased significantly (162 ± 112 to 146 ± 74 mg/dL, p = 0.02).

**Conclusion:**

Administration of probiotics to patients with advanced CKD was associated with decreases in syndecan-1 and blood glucose levels, indicating potential improvements in metabolism and decreased systemic inflammation.

## Resumo

**Introdução:**

A suplementação com probióticos na doença renal crônica (DRC) pode estar associada à redução do processo inflamatório sistêmico.

**Objetivo:**

Avaliar a suplementação oral com probióticos em pacientes com DRC em hemodiálise.

**Método:**

Ensaio clínico, duplo cego, randomizado com 70 pacientes em hemodiálise, sendo 32 do grupo que recebeu o suplemento de probióticos e 38 do grupo placebo. Inicialmente ocorreu a coleta de sangue e suplementação oral com probióticos ou placebo durante três meses. O suplemento probiótico foi composto pela combinação de 4 cepas de bactérias Gram-positivas encapsuladas: Lactobacillus Plantarum A87, Lactobacillus rhamnosus, Bifidobacterium bifidum A218 e Bifidobacterium longum A101, sendo 1 cápsula do suplemento ao dia, durante 3 meses. Após esse período foram feitas novas coletas de sangue para dosagem dos biomarcadores inflamatórios. Foram analisados os biomarcadores não tradicionais: Syndecan-1, IFN-y, NGAL e cistatina C pelo método ELISA, e os seguintes parâmetros bioquímicos: PCR, cálcio, fósforo, potássio, PTH, TGP, hematócrito, hemoglobina, glicose e ureia.

**Resultados:**

Os pacientes que receberam suplemento tiveram diminuição significativa dos níveis séricos de syndecan-1 (de 239 ± 113 para 184 ± 106 ng/mL, p = 0,005). Outro parâmetro que diminuiu significativamente nos pacientes que receberam suplemento foi a glicemia (de 162 ± 112 para 146 ± 74 mg/dL, p = 0,02). Conclusão: O uso de probióticos na DRC avançada esteve associado à redução dos níveis de syndecan-1 e glicemia, sinalizando possível melhora no metabolismo e redução do processo inflamatório sistêmico.

## Introduction

The incidence of chronic kidney disease (CKD) in Brazil has increased significantly. According to the 2020 Brazilian Dialysis Census, an estimated 144,779 individuals were on dialysis in the nation, in line with the increase seen in recent years^1^. An estimated 44,264 new patients sought care in dialysis centers in Brazil in 2020^1^.

CKD has been associated with a pro-inflammatory state, and the eating habits of individuals with CKD may be linked to such a state^2^. Patients with CKD on hemodialysis are more susceptible to gut dysbiosis, which increases the risk of complications in individuals with CKD and cardiovascular disease^3^. Use of probiotics may enrich the gut microbiota, enhance immune response, restore intestinal permeability, and promote anti-inflammatory effects^4^ by correcting dysbiosis, thus potentially producing beneficial effects for patients with CKD^3^. Recent literature reviews on the administration of probiotics to patients with CKD reported favorable effects, observed as decreases in the levels of biomarkers of oxidative stress (malondialdehyde), inflammation (interleukin-6), urea, p-cresol, ammonia, among other positive effects^5,6^.

Cystatin C and NGAL have been used mainly as early biomarkers of kidney injury, and carry an association with inflammation^7,8,9,10^. Recent studies have described decreases in traditional markers of inflammation such as C-reactive protein (CRP) in patients with CKD after the administration of probiotics, and improved kidney function evinced by decreases in cystatin C levels^5,11^.

This study aimed to evaluate whether the administration of probiotics to patients with CKD on hemodialysis might decrease systemic inflammation and contribute to improving patient metabolic profiles.

## Methods

### Study Design

This double-blind randomized clinical trial (RCT) enrolled 70 patients seen at a dialysis clinic in Fortaleza, Ceará, Brazil. Thirty-two were given supplementation with probiotics and 38 were administered placebo. The patients included in the trial signed an informed consent term. They were made aware of what joining the trial as a volunteer entailed in terms of collection of biological material and data from their medical charts.

### Participants

**Figure 1.**
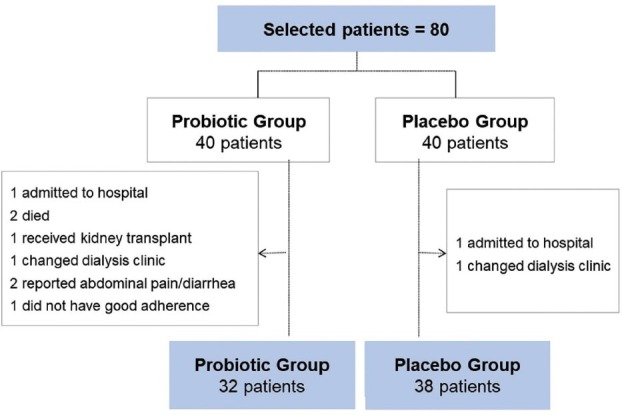
Study flowchart.

Female and male individuals aged between 22 and 69 years diagnosed with CKD and on hemodialysis were included in the trial. Patients already on probiotics were excluded, along with pregnant and possibly pregnant women; kidney transplant patients; subjects with gastrointestinal disorders including cancer; patients with a history of gastrointestinal surgery; individuals with behavioral disorders, due to difficulty obtaining answers in an interview; subjects with paraplegia, tetraplegia or amputations, due to altered anthropometric standards. Patients with other diseases such as HIV/Aids, tumors, and autoimmune disease were excluded, along with individuals on medications that might significantly interfere with test results or affect inflammation and the microbiota, such as antibiotics and anti-inflammatory drugs. Older patients were also excluded on account of the morphological and functional changes that come with aging, including lower kidney function, as previously described^12^. Children and adolescents were excluded for reasons tied to anthropometric categorization^13^. Patients unable to take probiotics regularly were also excluded. The inclusion and exclusion criteria are illustrated in Figure 1.

### Interventions

The supplements and placebo offered to patients had equal organoleptic characteristics, i.e., the capsules had the same color, physical appearance, and flavor. The quality of the capsules was assured by the compounding pharmacy. The probiotic supplement comprised four strains of encapsulated Gram-positive bacteria: Lactobacillus Plantarum A87, Lactobacillus rhamnosus, Bifidobacterium bifidum A218 and Bifidobacterium longum A101. Each strain was offered at a concentration of 1 billion colony-forming units (CFU), adding to a total of 4 billion CFU in each capsule. Patients took one capsule per day, after dinner, for three months. They were also advised about how to properly store the capsules. The formulation chosen for the trial was based on statements made by the ANVISA (Brazilian Health Surveillance Agency), which attested to the probiotic potential of genera Lactobacillus and Bifidobacterium, and due to the fact that they account for 80% of the microbiota of a healthy individual^14^.

During the intervention period, a team of five students from the UNIFOR Medical School followed the patients on a weekly basis via telephone calls, in order to monitor compliance to treatment and adverse events. Data was collected before the introduction of probiotics or placebo and after three months of treatment. Participants had blood samples taken before dialysis for the verification of non-traditional biomarkers of inflammation and were weighed dialysis. They were given containers with probiotics or placebo. After thee months of treatment, the same parameters measured and checked at the start of the trial were assessed again, following the same quality standards.

### Bmi Assessment

The formula (aBWef (18) = DBW (kg) + [(RBW – DBW) × 0,25]) was used to calculate the edema-free adjusted body weight, in which aBWef is edema-free adjusted body weight; DBW is dry body weight; and RBW is reference body weight, as set out in the Clinical practice guideline for nutrition in chronic renal failure: 2021^15^. The body mass index (BMI) was calculated using the formula Weight/(Height)^2^ defined by the World Health Organization in 1995 used as a reference to this day.

### Blood Sample Collection

Blood samples from the participants were collected before the start of dialysis in each respective shift. Blood was drawn into 5-mL Vacutainer® tubes with yellow caps containing separating gel. Each tube had been previously tagged with each participant's name. A shelf, Styrofoam boxes, and cooling packs were used to transport the blood samples to the laboratory. The blood stored in the tubes was centrifuged at 2500 revolutions per minute (RPM) for 10 minutes. A volumetric pipette was used to transfer the obtained plasma into 1.5 mL Eppendorf micro tubes; the aliquots were then stored at –80ºC until further analysis.

### Non-Traditional Inflammatory Markers

Non-traditional biomarkers (NGAL, Cystatin C, CRP, and IFN-y) were quantified from aliquots processed on the day of collection. ELISA was the method used for its high sensitivity and specificity. ELISA test kits from R&D Systems® (R&D Systems, Minneapolis, MN, USA) were procured, with enough reagent for five to fifteen 96-well plates depending on the biomarker. All tests were performed from serum samples.

### Endpoints

The main inflammatory metabolic and inflammatory parameters were assessed after three months of treatment. The primary endpoint was decreased inflammation and metabolic disorder derived from lower levels of biomarkers CPR, cystatin C, NGAL, and blood glucose.

### Randomization

Software program Research Randomizer® (www.randomizer.org) was used to randomly assign participants to study groups. Individuals in Group 1 (G1) were administered supplementation with probiotics and subjects in Group 2 (G2) were given placebo. Since this was a double-blind study, the researcher and the participants were blinded for the sequential allocation and the codes assigned to supplementation or placebo. The supplements and placebo offered to patients had equal organoleptic characteristics, i.e., the capsules had the same color, physical appearance, and flavor. The quality of the capsules was assured by the compounding pharmacy. Placebo comprised an inert allergen-, gluten-, soybean-, and lactose-free formulation without drug interactions, made from a base of modified microcrystalline cellulose known by the trade name Celulamax E®. Celulamax E® was chosen for meeting the criteria stipulated in health safety regulations^16,17^.

### Statistical Methods

Quantitative variables were expressed as mean values ± standard deviation based on data normality. The Shapiro-Wilk test of normality was used. Qualitative variables were expressed as absolute counts and proportions. The chi-squared test or Fisher's exact test was used in categorical data comparisons between independent groups. McNemar's test was used in comparisons of categorical data between dependent groups (before and after supplementation). Depending on data normality, Student's t-test or the Mann-Whitney U test was used in comparisons of quantitative data between independent groups in each time period (before and after supplementation). Statistical significance was attributed to differences with a P &lt; 0.05. Data sets were analyzed on SPSS for Macintosh, release 23 (SPSS, IBM, USA).

### Ethics Committee Approval

The Ethics Committee for Human Research of the University of Fortaleza (UNIFOR) approved this study (certificate no. 3325286/2019). The patients included in the study signed informed consent terms and were given a comprehensive explanation of the purpose and procedures involved in the study.

## Results

The present trial included 80 patients on hemodialysis (HD). Seventy completed the intervention protocol, 38 in the placebo and 32 in the supplementation group. Ten participants left the trial. Eight patients in the Group given supplementation with probiotics left the study for the following reasons: one patient was hospitalized; two died; one underwent kidney transplantation; one changed clinics; two reported abdominal pain and diarrhea; and one failed to comply with the protocol. In the Group given placebo, one patient was hospitalized and one changed clinics during the trial.

The baseline characteristics of the patients in the two groups were similar. PTH levels were the only significant difference found, with individuals in the group given supplementation presenting higher levels (Table 1).

**Table 1.** Overall and workup characteristics of study participants before the introduction of probiotics: a comparison between individuals in the placebo and probiotics groups

	Before administration of probiotics		p*
	Placebo (n = 38)	Probiotics (n = 32)	
**Sex**			0,602
Male	19 (50)	14 (43,8)	
Female	19 (50)	18 (56,3)	
**Age, years**	49 ± 13	47 ± 13
**BMI category**			0,479
Underweight	2 (5,3)	0 (0)	
Normal weight	29 (76,3)	23 (71,9)	
Overweight	6 (15,8)	8 (25)	
Obese	1 (2,6)	1 (3,1)	
**CRP (ng/mL)**	1563,3 ± 783	1626 ± 617,5	0,715
**Syndecan-1 (ng/mL)**	211,95 ± 121,5	239,48 ± 113,61	0,334
**IFN-y (detectable)**	7 (18,4)	1 (3,1)	0,063
**NGAL (ng/mL)**	59,02 ± 20,18	65,67 ± 12,45	0,110
**Cystatin C (ng/mL)**	713,2 (398,5 – 815,5)	620,4 (212,1 – 993,8)	0,084
**Calcium (mg/dL)**	8,6 ± 0,8	8,7 ± 0,5	0,551
**Phosphorus (mg/dL)**	5 ± 1,7	5,2 ± 1,2	0,531
**PTH (U/L)**	400,6 ± 253,7	738,6 ± 592,8	0,047
**GPT (U/L)**	12 (9 – 16)	12 (10 – 18)	0,714
**Ht (%)**	33,3 ± 5,4	33,5 ± 5,1	0,904
**Hb (g/dL)**	11 ± 1,9	11,1 ± 1,8	0,762
**K (mEq/L)**	4,7 ± 0,7	5,1 ± 1,3	0,145
**Glucose (mg/dL)**	151,5 ± 93,9	162,7 ± 112,6	0,761
**Urea – pre (mg/dL)**	114 ± 35,8	122,8 ± 30,6	0,303
**Urea – post (mg/dL)**	31 ± 14,2	32,5 ± 16,5	0,707
**BMI (kg/m^2^)**	23 ± 2,8	23,7 ± 2,6	0,352

CRP: C-reactive protein; IFN-y: interferon gamma; NGAL: neutrophil gelatinase-associated lipocalin; PTH: parathyroid hormone; GPT: glutamic pyruvic transaminase; Ht: Hematocrit; Hb: Hemoglobin; K: potassium; BMI: body mass index; Significant: p &lt; 0.05. Qualitative data expressed as absolute counts and proportions between brackets. Quantitative data expressed as mean values ± standard deviation or median and interquartile range between brackets. *Student's t-test or the Mann-Whitney U test was used to compare quantitative data and the chi-squared or Fisher's exact test was used to evaluate associations between qualitative data.

No significant difference was found in the comparison between the individuals given probiotics and the patients offered placebo at the end of the supplementation period (Table 2).

**Table 2.** Clinical characteristics at the end of the administration of probiotics

	After administration of probiotics		p*
	Placebo (n = 38)	Probiotics (n = 32)	
**BMI category**			0,479
Underweight	2 (5,3)	0 (0)	
Normal weight	29 (76,3)	23 (71,9)	
Overweight	6 (15,8)	8 (25)	
Obese	1 (2,6)	1 (3,1)	
**CRP (ng/mL)**	860 ± 331	898 ± 313	0,622
**Syndecan-1 (ng/mL)**	211,4 ± 198,1	184,3 ± 106	0,491
**IFN-y (detectable)**	6 (15,8)	4 (12,5)	0,695
**NGAL (ng/mL)**	60,5 ± 27,1	64,2 ± 25,8	0,571
**Cystatin C (ng/mL)**	527,35 (507,01 – 540,49)	537,89 (505,5 – 559,4)	0,358
**Calcium (mg/dL)**	10,9 ± 13,3	10,9 ± 12,7	0,988
**Phosphorus (mg/dL)**	5,4 ± 1,6	5 ± 1,3	0,299
**PTH (U/L)**	242,7 ± 177,4	677,1 ± 841,6	0,072
**GPT (U/L)**	11 (9 – 14)	11 (8 – 14)	0,669
**Hematocrit (%)**	35,2 ± 6,8	37,1 ± 5,4	0,254
**Hemoglobin (g/dL)**	11,5 ± 2,2	12 ± 1,5	0,29
**Potassium (mEq/L)**	5,1 ± 0,7	5,2 ± 1,4	0,785
**Glucose (mg/dL)**	156,9 ± 98,2	146,5 ± 74,6	0,766
**Urea – pre (mg/dL)**	111,5 ± 45,8	117,5 ± 28,9	0,553
**Urea – post (mg/dL)**	31,4 ± 14,7	34,2 ± 25,1	0,586
**BMI (kg/m^2^)**	23 ± 2,8	23,7 ± 2,6	0,325

CRP: C-reactive protein; IFN-y: interferon gamma; NGAL: neutrophil gelatinase-associated lipocalin; PTH: parathyroid hormone; GPT: glutamic pyruvic transaminase; Ht: Hematocrit; Hb: Hemoglobin; K: potassium; BMI: body mass index; Significant: p &lt; 0.05. Qualitative data expressed as absolute counts and proportions between brackets. Quantitative data expressed as mean values ± standard deviation or median and interquartile range between brackets. *Student's t-test or the Mann-Whitney U test was used to compare quantitative data and the chi-squared or Fisher's exact test was used to evaluate associations between qualitative data. Source: the author.

Matched analysis of all parameters between the two time periods (before vs. after supplementation) and groups (placebo vs. probiotics) found a significant association after three months between inflammatory mediators and use of probiotics.

In the placebo group, the levels of inflammatory mediator CRP decreased at the end of the trial (1563 ± 783 to 860 ± 331 ng/mL, p = 0.001). Other parameters did not differ statistically in this group when levels before and after treatment were compared (Table 3). In the group given probiotics, CRP levels also decreased significantly (1626 ± 617 to 898 ± 313 ng/mL, p = 0.001) when serum levels before and after supplementation were compared. Hemoglobin and hematocrit levels increased significantly in the group given probiotics (Table 4). In the group given probiotics, no significant decrease was observed in kidney parameters, including serum NGAL and cystatin C.

**Table 3.** Clinical and workup characteristics before and after administration of supplementation of subjects in the placebo group

	Placebo (n = 38)		p*
	Before supplementation	After supplementation	
**BMI category**			1,000
Underweight	2 (5,3)	1 (2,6)	
Normal weight	29 (76,3)	23 (60,5)	
Overweight	6 (15,8)	5 (13,2)	
Obese	1 (2,6)	10 (26,3)
CRP (ng/mL)	1563,3 ± 783	860 ± 331	0,001
**Syndecan-1 (ng/mL)**	211,95 ± 121,5	211,4 ± 198,1	0,984
**IFN-y (detectable)**	7 (18,4)	6 (15,8)	1,000
**NGAL (ng/mL)**	59,02 ± 20,18	60,5 ± 27,1	0,737
**Cystatin C (ng/mL)**	713,2 (398,5 – 815,5)	527,35 (507,01 – 540,49)	0,072
**Calcium (mg/dL)**	8,6 ± 0,8	10,9 ± 13,3	0,339
**Phosphorus (mg/dL)**	5 ± 1,7	5,4 ± 1,6	0,388
**PTH (U/L)**	400,6 ± 253,7	242,7 ± 177,4	0,17
**GPT (U/L)**	12 (9 – 16)	11 (9 – 14)	0,786
**Hematocrit (%)**	33,3 ± 5,4	35,2 ± 6,8	0,434
**Hemoglobin (g/dL)**	11 ± 1,9	11,5 ± 2,2	0,571
Potassium (mEq/L)	4,7 ± 0,7	5,1 ± 0,7	0,076
**Glucose (mg/dL)**	151,5 ± 93,9	156,9 ± 98,2	0,916
**Urea – pre (mg/dL)**	114 ± 35,8	111,5 ± 45,8	0,491
**Urea – post (mg/dL)**	31 ± 14,2	31,4 ± 14,7	0,452
**BMI (kg/m2)**	23 ± 2,8	23 ± 2,8	1,000

CRP: C-reactive protein; IFN-y: interferon gamma; NGAL: neutrophil gelatinase-associated lipocalin; PTH: parathyroid hormone; GPT: glutamic pyruvic transaminase; Ht: Hematocrit; Hb: Hemoglobin; K: potassium; BMI: body mass index; Significant: p &lt; 0.05. Qualitative data expressed as absolute counts and proportions between brackets. Quantitative data expressed as mean values ± standard deviation or median and interquartile range between brackets. *The paired t-test or Wilcoxon's test was used in comparisons featuring quantitative data and McNemar's test was used to assess the associations between qualitative data.

**Table 4.** Clinical and workup characteristics before and after administration of supplementation of subjects in the probiotics group

	Placebo (n = 32)		p*
	Before supplementation	After supplementation	
**BMI category**			1,000
Underweight	0 (0)	1 (3,1)	
Normal weight	23 (71,9)	10 (31,3)	
Overweight	8 (25)	1 (3,1)	
Obese	1 (3,1)	21 (65,6)	
**CRP (ng/mL)**	1626 ± 617,5	898 ± 313	0,001
**Syndecan-1 (ng/mL)**	239,48 ± 113,61	184,3 ± 106	0,005
**IFN-y (detectable)**	1 (3,1)	4 (12,5)	0,375
**NGAL (ng/mL)**	65,67 ± 12,45	64,2 ± 25,8	0,772
**Cystatin C (ng/mL)**	620,4 (212,1 – 993,8)	537,89 (505,5 – 559,4)	0,112
**Calcium (mg/dL)**	8,7 ± 0,5	10,9 ± 12,7	0,371
**Phosphorus (mg/dL)**	5,2 ± 1,2	5 ± 1,3	0,356
**PTH (U/L)**	738,6 ± 592,8	677,1 ± 841,6	0,421
**GPT (U/L)**	12 (10 – 18)	11 (8 – 14)	0,576
**Hematocrit (%)**	33,5 ± 5,1	37,1 ± 5,4	0,011
**Hemoglobin (g/dL)**	11,1 ± 1,8	12 ± 1,5	0,036
**Potassium (mEq/L)**	5,1 ± 1,3	5,2 ± 1,4	0,817
**Glucose (mg/dL)**	162,7 ± 112,6	146,5 ± 74,6	0,026
**Urea – pre (mg/dL)**	122,8 ± 30,6	117,5 ± 28,9	0,516
**Urea – post (mg/dL)**	32,5 ± 16,5	34,2 ± 25,1	0,802
**BMI (kg/m2)**	23,7 ± 2,6	23,7 ± 2,6	1

CRP: C-reactive protein; IFN-y: interferon gamma; NGAL: neutrophil gelatinase-associated lipocalin; PTH: parathyroid hormone; GPT: glutamic pyruvic transaminase; Ht: Hematocrit; Hb: Hemoglobin; K: potassium; BMI: body mass index; Significant: p &lt; 0.05. Qualitative data expressed as absolute counts and proportions between brackets. Quantitative data expressed as mean values ± standard deviation or median and interquartile range between brackets. *The paired t-test or Wilcoxon's test was used in comparisons featuring quantitative data and McNemar's test was used to assess the associations between qualitative data.

Blood levels of syndecan-1, a marker of endothelial glycocalyx, decreased significantly in patients given probiotics (239 ± 113 to 184 ± 106 ng/mL, p = 0.005) (Table 2). Blood glucose also decreased significantly in patients given probiotics (162 ± 112 to 146 ± 74 mg/dL, p = 0.02) (Table 2).

## Discussion

This study found an association between use of probiotics among patients with CKD on hemodialysis, decreased levels of endothelial lesion markers (syndecan-1), and improved lab parameters for blood glucose, hematocrit, and hemoglobin. Interest around probiotic supplements has grown in recent years, including in Brazil^18^, with new meta-analyses currently being prepared to assess the effects of probiotics on CKD^19^.

Early studies on gut microbiota and CKD did not find significant differences between the number of microorganisms in the microbiota of patients with CKD versus healthy individuals, although Bifidobacteria spp counts were lower and Escherichia coli, Klebsiella pneumoniae and Enterococcus were more prevalent in the GI tract of subjects with kidney disease^20^. Other studies indicated that the gut microbiota of individuals with CKD might be altered, presenting greater counts of potentially pathogenic and proinflammatory bacteria that contributed to the progression of kidney disease^21^.

The need to further confirm the alterations in individuals with CKD cited above prompted the organization of this trial, which focused on assessing the effects of supplementation with probiotics formulated with strains of Lactobacillus plantarum A87, Lactobacillus rhamnosus, Bifidobacterium bifidum A218, Bifidobacterium longum A101, at a total concentration of 4 CFU, on non-traditional biomarkers in patients with CKD on hemodialysis.

Our results showed that, after three months of treatment, significant decreases were observed in the levels of endothelial glycocalyx marker syndecan-1 and blood glucose, accompanied by significant increases in hemoglobin and hematocrit levels in the group given probiotics when compared to the placebo group. However, such findings cannot be attributed solely to probiotic supplementation, since patients on dialysis were also given therapy for anemia, a frequent complication in CKD.

Syndecan-1 is one of the main components of endothelial glycocalyx. Cleavage of is extracellular domains releases its plasma-soluble form^22,23^. Inflammatory stimuli or injury to the endothelial glycocalyx increase its expression in the immune system and release in serum, a condition present in individuals with CKD on hemodialysis^24^.

In line with the results presented in this trial concerning the significant decrease in histological scores for syndecan-1, Le et al.^25^ reported that the two genera more broadly studied and tied to decreases in gastrointestinal inflammation are Lactobacillus and Bifidobacteria. The two exhibited therapeutic effects against gastrointestinal inflammation by modulating against inflammatory cytokines. The decrease in syndecan-1 levels may serve as evidence of the effect of supplementation with probiotics at reducing endothelial inflammation, which indirectly decreases cardiovascular risk. Few studies have looked into the association between gut microbiota and syndecan-1, although evidence indicates that syndecan-1 plays an important role in intestinal ischemia/reperfusion injury^26^. More studies are needed to assess the systemic effects of supplementation with probiotics, particularly in relation to possible beneficial cardiovascular effects. Some authors have suggested that probiotics contribute to decrease and control dyslipidemia and decrease blood pressure and inflammatory mediators, among others^27^.

In regard to the association with lower blood glucose levels, Soleimani et al.^28^ also observed beneficial effects on glucose parameters and some biomarkers of inflammation when controls were compared to 60 patients with diabetes on hemodialysis given supplementation for 12 weeks with a formula containing Lactobacillus acidophilus, Lactobacillus casei and Bifidobacterium bifidum at a dose of 2 × 10^9^ CFU/g of each strain in a double-blind clinical trial.

No significant differences were found in non-traditional biomarkers NGAL, cystatin C, and inflammatory mediator IFN-y levels. A randomized clinical trial organized by Miraghajani et al.^29^ enrolled 60 patients with diabetic nephropathy to find the potential effects of drinking soy milk enriched with Lactobacillus plantarum A7 on novel renal factors NGAL e cystatin C. Case group members were asked to drink 200 mL/day of the probiotic preparation with soy milk, while controls had soy milk alone for eight weeks. Miraghajani et al.^29^ concluded that probiotic soy milk had a beneficial effect on kidney function, since it decreased NGAL (p = 0.05) and cystatin C (p = 0.02) levels. Cystatin C and NGAL are biomarkers of early kidney injury and inflammation, and cystatin C is also a marker of dialysis adequacy^7–10^. Therefore, the rationale behind the use of these biomarkers in this trial was to assess them as markers of inflammation and not of kidney function, since one of the main hypotheses concerning the effect of supplementation with probiotics is that it decreases systemic inflammation. However, no significant decrease in cystatin C and NGAL levels was observed after supplementation with probiotics in our trial.

A decrease was observed in CRP levels, in the placebo and supplementation groups. In a study with a similar design enrolling patients on dialysis, Ranganathan et al.^30^ did not find significant differences in parameters such as urea, creatinine, and CRP after two months of treatment. A triple-blind randomized placebo-controlled trial enrolling 54 patients with diabetes given a symbiotic supplement containing seven dry-frozen strains at concentrations of 2 billion CFU of Lactobacillus acidophilus, 7 billion CFU of L. casei, 1.5 billion CFU of L. rhamnosus, 2 billion CFU of L. bulgaricus, 2 billion UFC of Bifidobacterium breve, 2 billion UFC of B. longum, 2 billion CFU of Streptococcus thermophilus, and 100 mg of a fructo-oligosaccharide prebiotic described a significant decrease in CRP serum levels in the individuals administered the preparation when compared to the subjects given placebo^31^.

Toumi et al.^32^ looked into the potential effects of a preparation made with four live bacteria strains (L. acidophilus, L. plantarum, B. lactis, and B. breve) on an animal model. After induction and establishment of intestinal injury with dextran sulfate sodium (DSS), mice were fed a probiotic preparation via oral gavage. In the experiment, the plasma level of IFN-y in induced colitis decreased after supplementation with probiotics; in the present trial, however, a significant decrease was not observed.

Non-regenerative normocytic normochromic anemia is often observed in the hematopoietic system^33^. Different factors favor the development of anemia during progression to CKD, including reduced erythrocyte life span; reduced glutathione erythrocyte levels; folate and vitamin B deficiency caused by polyuria and insufficient intake of these nutrients; iron deficiency due to low iron intake and gut absorption and low iron production levels due to erythropoietin deficit linked to kidney mass reduction^34^.

In line with the results published in the present study concerning the increase seen in the levels of hemoglobin and hematocrit in individuals given probiotics, the experimental study organized by Sakai et al.^35^ with a control group found that administering a prebiotic supplement to gastrectomized rats stimulated the absorption of iron in the large intestine, albeit minimally. In another similar trial, Ohta et al.^36^ reported increases in hematocrit and hemoglobin levels after supplementation with prebiotics and prevention of anemia in rats.

Our study had some limitations: it was carried out with patients from a single dialysis center; the study population was small; enrolled patients were followed for a short period of time. Food intake was not analyzed, although it might interfere with the gut microbiota and affect study results. However, our findings favor supplementation with probiotics to this group of patients.

In conclusion, the use of probiotics by patients with advanced CKD was associated with decreases in the levels of syndecan-1 and blood glucose, indicating a potential improvement in metabolism and decreased systemic inflammation. Since these findings were derived from a small patient population, more advanced statistical analyses such as logistic regression were not performed, nor was it possible to analyze the effect of potential confounding factors. Studies with a longer follow-up period including patients with other stages of CKD are needed to better recognize the effects of probiotics on individuals with kidney disease.
